# Changes in Primary Care Practice Setting and Practice Type for Medicare Beneficiaries

**DOI:** 10.1001/jamahealthforum.2025.0445

**Published:** 2025-04-25

**Authors:** Amelia M. Bond, William L. Schpero, Yasin Civelek, Kayla N. Tormohlen, Lawrence P. Casalino, David J. Jones, Manyao Zhang, Reekarl Pierre, Dhruv Khullar

**Affiliations:** 1 Division of Health Policy and Economics, Department of Population Health Sciences, Weill Cornell Medical College, New York, New York; 2 Cornell Health Policy Center, New York, New York; 3 Mathematica Policy Research, Cambridge, Massachusetts; 4 Division of General Internal Medicine, Department of Medicine, Weill Cornell Medical College, New York, New York; 5 The Physicians Foundation Center for the Study of Physician Practice and Leadership, Weill Cornell Medicine, New York, New York

## Abstract

This cross-sectional study examined changes in practice setting and practice type in 2012 vs 2022 among patients with traditional Medicare coverage.

## Introduction

Primary care is the foundation of good-quality health care, and where patients receive services (ie, practice setting and practice type) can affect health and financial outcomes.^1,2^ Recent health system changes, including new payment models and increased consolidation, have likely affected care delivery,^3^ but how practice setting and practice type have changed is not well understood. We examined changes in primary care visits by practice setting and practice type for traditional Medicare beneficiaries between 2012 and 2022.

## Methods

Using a 20% sample of Medicare Carrier and Outpatient claim files, we identified all primary care visits billed by primary care physicians (PCPs) or delivered by any clinician in federally qualified health centers (FQHCs) or rural health clinics (RHCs) in 2012 and 2022. The Weill Cornell Medical College Institutional Review Board deemed this cross-sectional study exempt because it used secondary data of deidentified research participants. We followed the STROBE reporting guideline.

Visits billed by PCPs were defined following methods from the Medicare Comprehensive Primary Care Plus program (except telehealth visits were included and home health services were excluded). Visits delivered in FQHCs and RHCs were identified following methods from the Medicare Shared Savings Program. PCPs were defined using Medicare Data on Provider Practice and Specialty files as physicians in general practice, family practice, geriatrics, or internal medicine (excluding hospitalists).

We examined changes in practice setting by determining the percentage of primary care visits across different places of service in each year (2012 and 2022). For services billed by PCPs, we examined changes in practice type by determining the share of visits in practices with varying percentages of PCPs compared with the total number of physicians in the practice. To account for possible compositional changes in the traditional Medicare population due to increasing Medicare Advantage penetration, we repeated the analyses after reweighting the 2022 sample to match the clinical and sociodemographic characteristics of the 2012 sample (eMethods in Supplement 1). Analyses were performed from October 2024 to January 2025 using Stata, version 18 (StataCorp).

## Results

**Figure 1.**
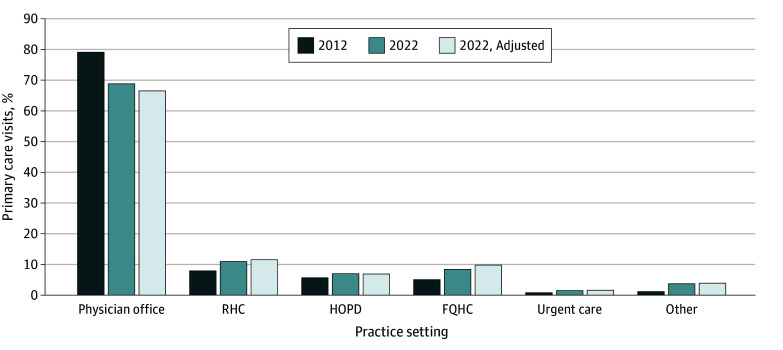
Percentage of Primary Care Visits With Primary Care Physicians or Safety Net Clinicians by Practice Setting in 2012 and 2022 Practice setting was defined by the place of service in Carrier claim files or revenue center codes or bill type in Outpatient claim files. The adjusted 2022 percentage represents a reweighted 2022 sample of primary care visits aligned with the 2012 sample, based on patient sex, age, hierarchical condition category risk score, and low-income subsidy eligibility status under Medicare Part D. FQHC indicates federally qualified health center; HOPD, hospital outpatient department; and RHC, rural health center.

**Figure 2.**
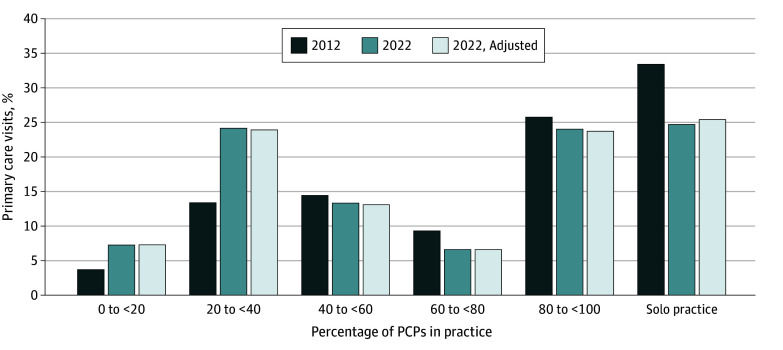
Percentage of Primary Care Visits by Percentage of Primary Care Physicians (PCPs) in Practice in 2012 and 2022 The adjusted 2022 percentage represents a reweighted 2022 sample of primary care visits aligned with the 2012 sample, based on patient sex, age, hierarchical condition category risk score, and low-income subsidy eligibility status under Medicare Part D. Practice type was determined by the percentage of PCPs vs the total number of physicians in the practice.

The sample included 5 100 348 Medicare beneficiaries (mean [SD] age, 71.8 [12.2] years; 2 962 340 female [58.1%]) with 22 087 860 primary care visits in 2012 and 4 526 754 beneficiaries (mean [SD] age, 73.3 [10.3] years; 2 588 941 female [57.2%]) with 16 932 935 visits in 2022. Between 2012 and 2022, the share of visits delivered in physician offices decreased from 79.2% to 68.8% and increased in RHCs (8.0% to 11.0%), FQHCs (5.2% to 8.2%), and hospital outpatient departments (HOPDs; 5.8% to 6.9%) (Figure 1). The specialty mix of practice settings also changed: the share of visits delivered by solo PCPs decreased from 33.4% to 24.7%, while the share delivered in practices with 20.0% to 39.9% PCPs increased from 13.4% to 24.2% (Figure 2). Analyses using the reweighted 2022 population found similar results.

## Discussion

Between 2012 and 2022, primary care visits for patients with traditional Medicare shifted from physician offices to FQHCs, RHCs, and HOPDs and from solo practices to multispecialty practices. Several policies may have contributed to these changes. The 2010 Affordable Care Act made investments in FQHCs and RHCs to expand access to care. The law also spurred payment reforms that potentially played a role in mergers of small practices with hospitals or large multispecialty practices.^4^ Additionally, Medicare pays higher rates to HOPDs than independent physician offices, which likely facilitated a shift toward HOPDs and increased spending.^2^

Study limitations included the use of Tax Identification Numbers to categorize practices (which can represent billing and other administrative relationships rather than relevant group practice settings) and an inability to reliably capture care delivered by advanced practice clinicians (although analyses of states with more vs less restrictive scope-of-practice laws yielded similar patterns). Nonetheless, this study provides evidence of shifting primary care practice setting and practice types. Future research should examine the implications for health quality and spending.

## Notes

Supplement 1.
eMethods

Supplement 2.
Data Sharing Statement
