# Supplementary material for: Changes in Primary Care Practice Setting and Practice Type for Medicare Beneficiaries
Source: JAMA Health Forum. 2025 Apr 25;6(4):e250445. doi: 10.1001/jamahealthforum.2025.0445 (PMC12032562; doi:10.1001/jamahealthforum.2025.0445)
Supplement: Supplement 1. — eMethods [file jamahealthforum-e250445-s001.pdf]

Supplementary Online Content

Bond AM, Schpero WL, Civelek Y, et al. Changes in primary care practice setting and practice type for Medicare beneficiaries. *JAMA Health Forum.* Published online April 25, 2025. doi:10.1001/jamahealthforum.2025.0445

eMethods.

This supplementary material has been provided by the authors to give readers additional information about their work.

© 2025 Bond AM et al. *JAMA Health Forum*

eMethods

In this study, we used national Medicare Fee-for-Service Carrier and Outpatient claims data, as well as the Medicare Data on Provider Practice and Specialty (MD-PPAS) file. Primary care encounters were identified in the Carrier file, except those delivered at rural health clinics (RHCs), federal qualified health centers (FQHCs), critical access hospitals, and elective teaching amendment (ETA) hospitals, which were identified in the Outpatient file. Primary care visits delivered in physician offices included some visits with physicians who were employed by hospitals, but not working in hospital outpatient departments (HOPDs).

To classify Carrier claims as primary care services, we used the Current Procedural Terminology (CPT) codes reported in claims, following logic used by the Centers for Medicare and Medicaid Services (CMS) in the Comprehensive Primary Care Plus (CPC+) program.[1] We applied several modifications to the CPC+ logic. First, we excluded home health services. Second, we included telehealth services (CPT codes 99441-99443). Finally, we restricted services to those billed by (PRF_PHYS_NPI) primary care physicians, defined in MD-PPAS as general practice, family practice, geriatrics, or internal medicine physicians (excluding hospitalists and sub-specialists).

For RHCs, FQHCs, critical access hospitals, and ETA hospitals, we followed CMS's logic for the Medicare Shared Savings Program (MSSP).[2] Following MSSP logic, all services delivered to RHCs and FQHCs were considered primary care services, while only services delivered by primary care physicians at critical access hospitals and ETA hospitals were considered primary care services. The set of ETA hospitals were identified each year using Medicare's Healthcare Provider Cost Reporting Information System.

A service's location was determined using the place of service in Carrier claims or revenue center codes or bill type in Outpatient claims. We attributed primary care physicians to practices using their primary tax identification number (TIN) in the MD-PPAS file. Practices were categorized into quintiles based on the proportion of primary billing physicians who identified as primary care physicians or as solo primary care practices.

To construct the adjusted 2022 proportion of primary care visits by practice setting and practice type, we reweighted the 2022 sample of primary care visits to align with the 2012 sample along demographic and clinical dimensions. Specifically, we reweighted based on patient sex, age (five categories), Low-Income Subsidy eligibility, and concurrent hierarchical condition category risk score (based on 2012 quintiles).

---

[1] CPC+ Payment and Attribution Methodologists for Program Year 2021, Version 2. March 23, 2021. https://www.cms.gov/priorities/innovation/media/document/cpc-plus-payment-methodology-cy2021
[2] Medicare Shared Savings Program. Shared Savings and Losses and Assignment Methodology. Version #9. Appendix D. February 2021. https://www.cms.gov/files/document/medicare-shared-savings-program-shared-savings-and-losses-and-assignment-methodology-specifications.pdf-0

© 2025 Bond AM et al. *JAMA Health Forum*
