# Supplementary material for: Changes in Primary Care Practice Setting and Practice Type for Medicare Beneficiaries
Source: JAMA Health Forum. 2025 Apr 25;6(4):e250445. doi: 10.1001/jamahealthforum.2025.0445 (PMC12032562; doi:10.1001/jamahealthforum.2025.0445)
Supplement: Supplement 2. — Data Sharing Statement [file jamahealthforum-e250445-s002.pdf]

Data Sharing Statement

Bond. Changes in Primary Care Practice Setting and Practice Type for Medicare Beneficiaries. *JAMA Health Forum*. Published April 25, 2025. doi:10.1001/jamahealthforum.2025.0445

Data
Data available: No

Additional Information
Explanation for why data not available: Medicare data under DUA
